# Similar compounds versus similar conformers: complementarity between PubChem 2-D and 3-D neighboring sets

**DOI:** 10.1186/s13321-016-0163-1

**Published:** 2016-11-04

**Authors:** Sunghwan Kim, Evan E. Bolton, Stephen H. Bryant

**Affiliations:** National Center for Biotechnology Information, National Library of Medicine, National Institutes of Health, Department of Health and Human Services, 8600 Rockville Pike, Bethesda, MD 20894 USA

**Keywords:** PubChem, PubChem3D, Molecular similarity, Neighboring, Neighbor Preference Index (NPI)

## Abstract

**Background:**

PubChem is a public repository for biological activities of small molecules. For the efficient use of its vast amount of chemical information, PubChem performs 2-dimensional (2-D) and 3-dimensional (3-D) neighborings, which precompute “neighbor” relationships between molecules in the PubChem Compound database, using the PubChem subgraph fingerprints-based 2-D similarity and the Gaussian-shape overlay-based 3-D similarity, respectively. These neighborings allow PubChem to provide the user with immediate access to the list of 2-D and 3-D neighbors (also called “Similar Compounds” and “Similar Conformers”, respectively) for each compound in PubChem. However, because 3-D neighboring is much more time-consuming than 2-D neighboring, how different the results of the two neighboring schemes are is an important question, considering limited computational resources.

**Results:**

The present study analyzed the complementarity between the PubChem 2-D and 3-D neighbors. When all compounds in PubChem were considered, the overlap between 2-D and 3-D neighbors was only 2% of the total neighbors. For the data sets containing compounds with annotated information, the overlap increased as the data sets became smaller. However, it did not exceed 31% and substantial fractions of neighbors were still recognized by either PubChem 2-D or 3-D similarity, but not by both. The Neighbor Preference Index (NPI) of a molecule for a given data set was introduced, which quantified whether a molecule had more 2-D or 3-D neighbors in the data set. The NPI histogram for all PubChem compounds had a bimodal shape with two maxima at NPI = ±1 and a minimum at NPI = 0. However, the NPI histograms for the subsets containing compounds with annotated information had a greater fraction of compounds with a strong preference for one neighboring method to the other (at NPI = ±1) as well as compounds with a neutral preference (at NPI = 0).

**Conclusion:**

The results of our study indicate that, for the majority of the compounds in PubChem, their structural similarity to other compounds can be recognized predominantly by either 2-D or 3-D neighborings, but not by both, showing a strong complementarity between 2-D and 3-D neighboring results. Therefore, despite its heavy requirements for computational resources, 3-D neighboring provides an alternative way in which the user can instantly access structurally similar molecules that cannot be detected if only 2-D neighboring is used.

**Graphical Abstract:**
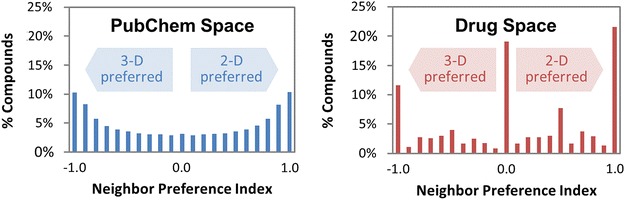
The binned distribution of the neighbor preference indices (NPIs) for all compounds in PubChem (*left*) has a bimodal shape with two maxima at NPI = ±1 and a minimum at NPI = 0, indicating that structural similarity between compounds in PubChem can be recognized predominantly by either 2-D or 3-D neighborings, but not by both. The NPI histogram for the drug space (*right*) has a greater fraction of compounds with a strong preference for one neighboring method to the other (at NPI ≈ ±1) as well as compounds with a neutral preference (at NPI ≈ 0), indicating that the drug space is very different from the PubChem space.

## Background

The rapid growth of information on chemicals and their biological activities has created a demand in the scientific community for public repositories that can increase the utility of these data, by collecting, integrating, and disseminating it to the community free of charge. An example of such repositories is PubChem [1–5], developed and maintained by the National Center for Biotechnology Information (NCBI), a part of the U.S. National Institutes of Health’s (NIH) National Library of Medicine (NLM). PubChem consists of three primary databases: Substance, Compound, and BioAssay. As of May 2016, 416 PubChem data contributors provide a wide range of chemical substance descriptions to the PubChem Substance database (accession: SID). The set of unique chemical structures present in the Substance database make up the PubChem Compound database (accession: CID). Results from biological experiments performed on the substance samples are stored in the PubChem BioAssay database (accession: AID). Altogether, PubChem is a sizeable data system of more than 219 million substance descriptions, 89 million unique chemical structures, one million biological assays, and 230 million biological assay outcomes (where an outcome is the set of results from a substance being tested in an assay).

There is a large variation in the amount of information available for each molecule contained in PubChem. For example, whereas some molecules have enormous quantities of information on the biological activity and literature associated, other molecules have very little information other than the chemical structure. When there is no desired information available for a particular molecule, one may infer it from its structural analogues that have relevant information. Even when a molecule has desired information, comparison with other available information on the molecule and its structural analogues may provide additional important insight.

To help find and analyze related information using chemical structures, PubChem provides services that exploit chemical structure similarity between molecules. These include Structure Search, Structure Clustering, and Structure–Activity Analysis [1, 6]. In addition, the PubChem Compound database provides two precomputed chemical structure similarity searches of molecules, dubbed “neighbor” relationships (where only results above a given threshold are retained from the similarity search). These give users immediate access to a set of structurally similar molecules. One of these neighboring relationships, known as “Similar Compounds”, uses the notion of 2-D similarity (which considers the atoms in a molecule and how they connect to each other) and is adept at finding close structural analogues of a structure such as those with the same scaffold. Another neighboring relationship in PubChem, known as “Similar Conformers”, uses the notion of 3-D similarity (which considers the overall shape and macromolecule-binding features of the molecule) [6, 7] and is adept at finding related structures with different scaffolds. As described in more detail in the “Methods” section, the 2-D neighboring uses the PubChem substructure fingerprint [8] and Tanimoto equation [9–11] to evaluate structural similarity between two molecules, resulting in a list of 2-D “Similar Compound” neighbors for each compound record. The Gaussian-shape overlay method by Grant and Pickup [12–15], which is implemented in the Rapid Overlay of Chemical Structures (ROCS) [16, 17], is used to generate a list of 3-D “Similar Conformer” neighbors for each molecule covered by the PubChem3D project [6, 7, 18–23]. This project generates 3-D conformer models for about 90% of the chemicals in the PubChem Compound database, being only those structures with a single component (i.e., no mixtures or salts), comprised of organic elements, not too flexible (≤15 rotatable bonds), and not too large (≤50 non-hydrogen atoms) [6, 18, 23]. These computationally derived conformer models are used in various PubChem tools and services that exploit 3-D similarity, including the 3-D conformer search, 3-D neighboring, 3-D clustering, 3-D structure–activity relationship analysis, and so on.

In general, binary fingerprint-based 2-D similarity methods can compare on the order of one million *compound* pairs per second per CPU core, but many 3-D similarity methods (such as ROCS [16, 17], used by PubChem) can only compare on the order of 100 ~ 1000 *conformer* pairs per second per CPU core. The CPU-based PubChem 3-D neighboring approach, as described in a recent study [20], uses various filtering schemes to preclude conformer pairs that cannot be neighbors of each other from the most time-consuming, shape superposition optimization step. As a result, the throughput of PubChem 3-D neighboring is enhanced beyond 100,000 *conformer* pairs per second per CPU core. It is important to note that GPU-based approaches show great promise to reduce the cost of 3-D similarity computation [24]. GPU implementations (such as FastROCS) provide on the order of 1,000,000 conformer pairs per second per GPU. The CPU-based filtering approach that accelerates PubChem 3-D neighboring can complement a GPU approach, where the CPU handles the filtering steps and the GPU perform the superposition optimization. However, if the 3-D neighboring considers ten diverse conformers per compound, a throughput of 100,000 *conformer pairs* per second per CPU core corresponds to a throughput on the order of magnitude of 1000 *compound pairs* per second per CPU core because there can be 100 conformer pairs (10 × 10 = 100) for each compound pair. Therefore, even though vastly accelerated, PubChem 3-D neighboring is still significantly slower than PubChem 2-D neighboring by three orders of magnitude.

One may legitimately ask the question, if 3-D neighboring is so computationally demanding, is there sufficient benefit to justify the additional computational effort over use of 2-D similarity? For example, how different are the results from 2-D and 3-D neighboring approaches? Do 2-D and 3-D similarity methods for a given chemical structure give unique chemical lists or do the two approaches largely approximate each other? What does 3-D similarity yield that 2-D similarity does not and vice versa? Are key molecules missed by one approach yet found by the other? The present study explores these questions by analyzing the overlap of 2-D and 3-D neighbors that are precomputed and stored in (and readily downloadable from) PubChem.

## Results and discussion

### Two series of data sets

As described in the “Methods” section, the present study compares PubChem 2-D and 3-D neighbors for ten different data sets (five data sets for each of two series A and B). The five sets in Series A are: (1) all chemicals in PubChem Compound, (2) just those chemicals with biomedical literature annotation (via MeSH [25]), (3) just those chemicals with macromolecule-bound experimental 3-D structure annotation (Protein Data Bank [26] ligands via the NCBI Molecular Modeling Database (MMDB) [27]), (4) just those chemicals with pharmacological action annotation (via MeSH, indicating a biological role is known), and (5) just those chemicals with drug annotation (via DailyMed [28], covering active ingredients in FDA approved drugs). These five sets with differing annotation type, designated as “PubChem-(A)”, “MeSH-(A)”, “Protein3D-(A)”, “PharmAct-(A)”, and “Drug-(A)”, respectively, can be obtained using the NCBI Entrez interface for the PubChem Compound database (see Table 1). Note that, by definition, the MeSH-(A), Protein3D-(A), PharmAct-(A), and Drug-(A) sets are all subsets of the PubChem-(A) set. Furthermore, PharmAct-(A) is a subset of MeSH-(A) and designates chemicals with a known biological role or activity. All PubChem 2-D and 3-D neighbors can be found on the PubChem FTP site (ftp://ftp.ncbi.nlm.nih.gov/pubchem/RDF/compound/).

**Table 1:** Number of compounds (CIDs) in the data sets employed in the present study

	Associated filters^a^	Series A	Series B	Ratio (B/A) (%)
PubChem	all	36,017,715	31,776,025	88.2
MeSH	pccompound_mesh	82,446	62,217	75.5
Protein3D	pccompound_structure	22,753	17,387	76.4
PharmAct	pccompound_mesh_pharm	11,415	6977	61.1
Drug	pccompound_drugs	1773	950	53.6

The five data sets in Series A were generated using associated Entrez filters, which are used to restrict a search to a particular compound subset in PubChem. The five data sets in Series B were generated from their Series A counterparts by adding the parent compounds of the chemicals in the Series A data sets and then selecting those with a computed 3-D conformer description available

^a^PubChem Compound Entrez filters allow users to retrieve CIDs that have a particular annotation type. For example, CIDs with “Drug” annotation can be retrieved via the URL: https://www.ncbi.nlm.nih.gov/pccompound/?term=pccompound_drugs[filter]

**Fig. 1:**
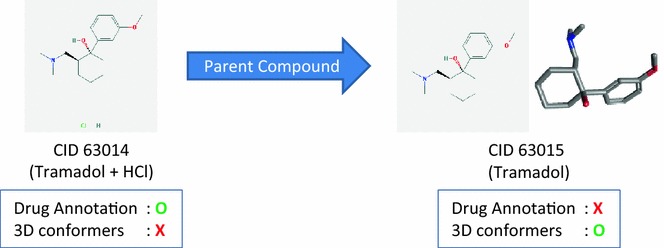
An inherent bias towards 2-D neighboring. CID 63014, a mixture of Tramadol (CID 63015) and HCl, has a drug annotation, but does not have a computed 3-D conformer description in PubChem. As such, CID 63014 cannot have any 3-D neighbors. On the contrary, its parent compound (CID 63015) has a computed 3-D conformer description, and therefore is able to have 3-D neighbors, but does not have a drug annotation. Use of CID 63015 (parent compound) in place of CID 63014 (salt mixture) allows 2-D salt forms to collapse into a single parent and 3-D neighboring methodologies to be compared (conceptually) for the same structure

 A direct comparison of 2-D and 3-D neighbors in PubChem may add an inherent bias for 2-D neighbors over 3-D neighbors as some chemicals do not have a computed 3-D conformer description [6, 18, 23] and hence cannot have 3-D neighbors. Examples are chemicals with multiple covalent units, as illustrated in Fig. 1. These chemicals are commonly found in the Drug-(A) set, because drugs are typically formulated as salt forms of the active pharmaceutical ingredient or as mixtures of the active (and inactive) ingredients. Note that various salt and mixture forms of the same active ingredient are likely to be highly similar to each other in terms of 2-D similarity, intensifying a 2-D neighboring bias. Therefore, for direct comparison purposes, this bias was removed by collapsing these multiple salt and mixture forms into their active ingredient, which conceptually corresponds to the “parent” component of a multi-component chemical structure (see the “Methods” section for the definition of a parent compound).

To remove the 2-D bias, the five sets in Series B [designated as PubChem-(B), MeSH-(B), Protein3D-(B), PharmAct-(B), and Drug-(B)] were generated by using the unique set of parent compound representations in the respective sets in Series A and then excluding any compound without a computationally generated PubChem3D conformer description. The Series B data sets ensure all considered chemicals have *both* 2-D and 3-D descriptions and also removes any redundancy due to salt/mixture form variation of the same parent chemical structure. Therefore, the Series B data sets would address any potential (2-D) bias, allowing the two neighboring approaches to be compared in an even way. The Series A data sets are also retained and analyzed for comparison purposes. Table 1 summarizes the number of compounds in each compound set. It is noteworthy that the Series B data sets consistently have fewer CIDs than their Series A counterparts, ranging from 11.8% fewer for the PubChem-(B) set to 46.4% fewer for the Drug-(B) set, further emphasizing the importance of bias removal for comparison purposes.

### Overlap between 2-D and 3-D neighbors in series A

**Fig. 2:**
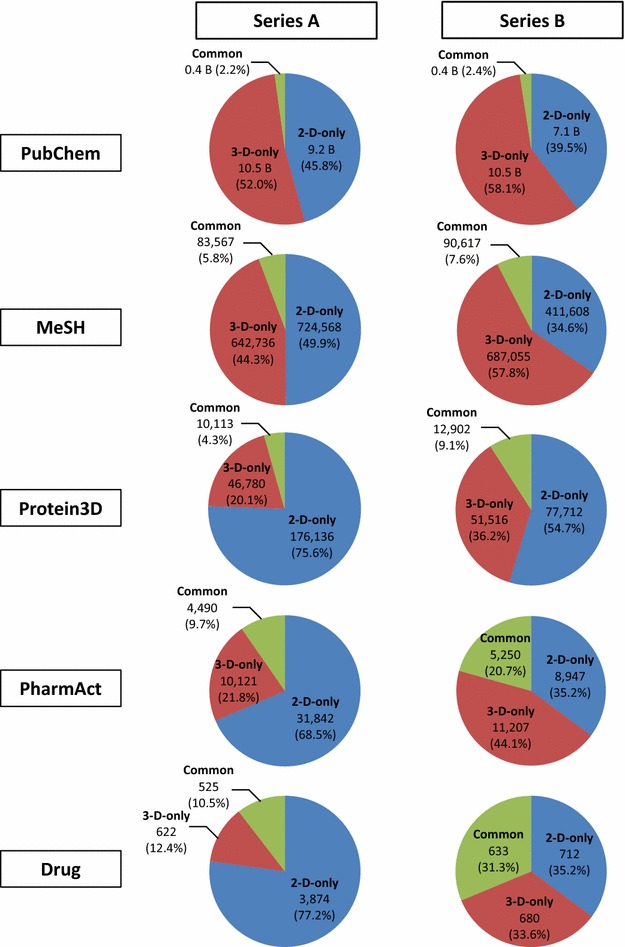
Comparison of 2-D and 3-D neighbor overlap. Each pie chart indicates the count and percentage of compound neighbor pairs as a function of data set and overlap. Series A contains all structures in PubChem, while Series B uses the parent compound of salts and is restricted to just those structures with a computed 3-D description (see Table 1)

Figure 2 shows the unique count of compound pairs that are neighbors of each other (simply referred to as “neighbor pairs” or “neighbors” hereafter) within the confines of each data set. For the PubChem-(A) set, there were 9.2 billion “2-D-only” neighbor pairs, recognized only by PubChem 2-D similarity, and 10.5 billion “3-D-only” neighbor pairs, recognized only by PubChem 3-D similarity. Interestingly, only 2.2% (0.4 billion pairs) of all PubChem-(A) neighbors were “common” neighbors, recognized by both 2-D and 3-D similarities. This rather small overlap between 2-D and 3-D neighbor sets indicates that the two similarity schemes are virtually orthogonal, with the structural similarity they recognize being very different.

**Fig. 3:**
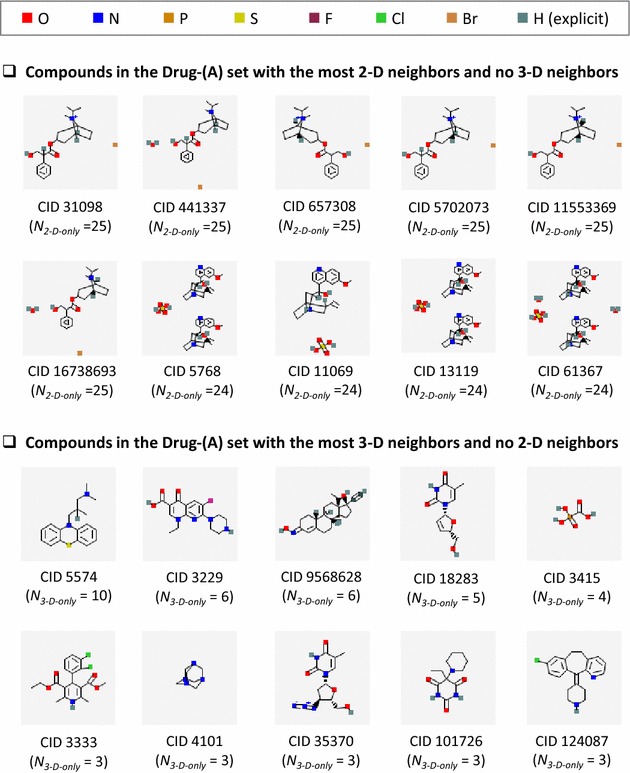
Examples of compounds in the Drug-(A) set that have a strong preference for one neighboring method over the other. Compounds in the Drug-(A) set with the most 2-D neighbors and no 3-D neighbors and compounds with the most 3-D neighbors and no 2-D neighbors. Note that all the compounds with 2-D-only neighbors have multiple covalent units

As shown in Fig. 2, the overlap between 2-D and 3-D neighbors increased as the size of the data sets decreased. However, even for the Drug-(A) set, which was the smallest in Series A, the overlap was only about 11% of all neighbors. Structural similarity for the remaining 89% can be recognized by only one of the two neighboring methods. Importantly, for the Drug-(A) set, there were much more “2-D-only” neighbor pairs than “3-D-only” neighbor pairs [77 vs. 12% of all neighbors in Drug-(A)], whereas they were comparable for the PubChem-(A) set [46 vs. 52% of all neighbors in PubChem-(A)]. This 2-D neighbor predominance in the Drug-(A) set appears to be primarily due to the bias for 2-D neighboring, as mentioned in the previous section and depicted in Fig. 1. This bias is also well illustrated in Fig. 3, which lists the top ten compounds in the Drug-(A) set with the most 2-D-only neighbors but without any 3-D neighbors. Note that all ten compounds are multi-component salt forms of nearly the same active ingredient.

### Overlap between 2-D and 3-D neighbors in Series B

The data sets in Series B remove salts and mixture forms that cause an inherent bias in favor of 2-D neighboring. In addition, only parent compounds that have computationally generated 3-D structures were considered. As a result, all five data sets in Series B were found to have considerably fewer 2-D neighbor pairs than in the Series A data sets (see Fig. 2). Similarly to Series A, the fraction of common neighbor pairs in the Series B data sets increased as the size of the data set decreased. However, for the smallest set, Drug-(B), the overlap between the 2-D and 3-D neighbor pairs was still only 31% and structural similarity of the remaining 69% was recognized only by one of the two neighboring approaches. The proportions of the 2-D-only and 3-D-only neighbor pairs are very close to each other (35 and 34%, respectively) for the Drug-(B) set, suggesting that the two approaches used by PubChem are complementary.

**Fig. 4:**
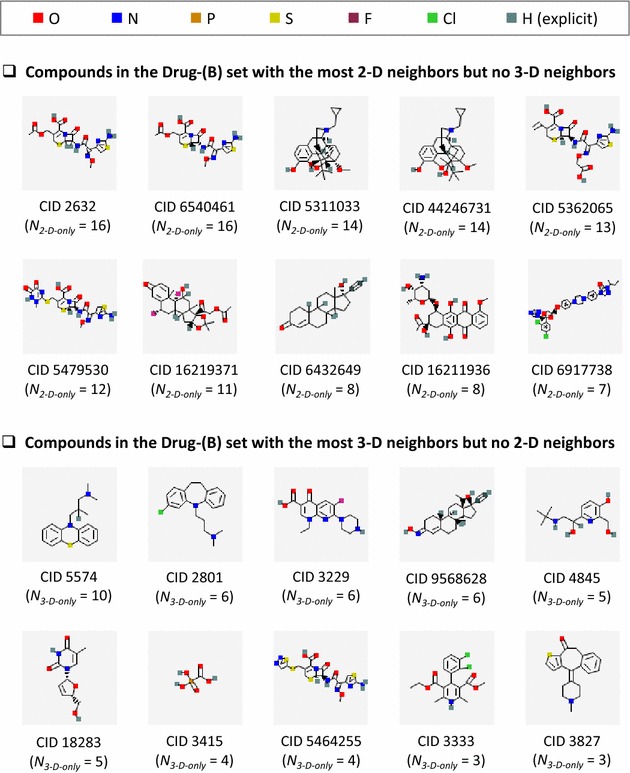
Examples of compounds in the Drug-(B) set that have a strong preference for one neighboring method over the other. Compounds in the Drug-(B) set with the most 2-D neighbors and no 3-D neighbors and compounds with the most 3-D neighbors and no 2-D neighbors. Note that all the compounds with 2-D-only neighbors differ considerably from those in Fig. 3, whereas most of the 3-D-only neighbors are nearly the same

Figure 4 illustrates the top ten Drug-(B) compounds with the most 2-D-only neighbors but without 3-D neighbors and those with the most 3-D-only neighbors but without 2-D neighbors. Interestingly, some of these compounds have common structural characteristics. For example, four of the ten compounds with the most 2-D-only neighbors (CID 2632, CID 6540461, CID 5362065, and CID 5479530) are cephalosporins, which is a class of *β*-lactam antibiotics. Indeed, three of the four have a pharmacological action annotation, “Anti-Bacterial Agent”. CID 5311033 and CID 44246731 have the same connectivity as each other, but the latter does not have explicitly defined stereocenters. On the other hand, the top ten compounds with the most 3-D-only neighbors in Fig. 4 [for the Drug-(B) set] are nearly unchanged from Fig. 3 [for the Drug-(A) set]. Therefore, it is reasonable to ask whether each compound in PubChem has an inherent preference for one neighboring method over the other. In other words, can structural similarity of a given compound with other molecules be recognized by only one PubChem similarity method, and not by the other for whatever reason?

### Distribution of neighbor preference indices (NPIs)

To determine the extent to which chemicals in PubChem can be interrelated by one similarity method but not the other, the Neighbor Preference Index (NPI) of a compound was introduced. It was designed to measure the extent of overlap between PubChem 2-D and 3-D neighboring approaches. If 2-D neighboring results substantially overlap with 3-D neighboring results, there would be little point to compute 3-D similarity, which is computationally expensive. As defined in the “Methods” section, this NPI quantity may have any value ranging from −1 (for compounds with 3-D neighbors only) to +1 (for compounds with 2-D neighbors only). A compound that has an equal number of 2-D and 3-D neighbors has an NPI value of zero, indicating that it has no preference for any of the two neighboring methods. The NPI value of a compound is dependent on the nature of the given chemical set, because a compound can have different sets of neighbors for different data sets.

**Fig. 5:**
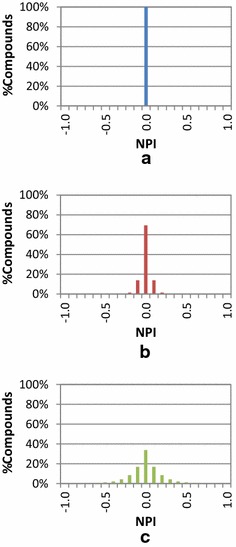
Hypothetical distributions of the neighboring preference indices (NPIs). The distribution of the NPI values of compounds in a data set under three hypothetical scenarios in which the two neighboring methods **a** are exactly identical, **b** are not exactly identical but are still very similar, and **c** become less and less similar to each other

One can imagine three hypothetical scenarios concerning the distribution of the NPI values of all compounds in a given data set, as illustrated in Fig. 5. First, if 2-D and 3-D similarity methods are identical and give exactly the same sets of neighbors as each other, the NPI values of all compounds will be zero, and the histogram of the NPI values will have a single column at NPI = 0 [Panel (a) in Fig. 5]. Second, if the two similarity methods are not exactly identical, but still somewhat similar to each other, the neighbor lists from the two methods for a given compound will have a substantial number of common neighbors. Therefore, the resulting NPI histogram will still have a maximum at NPI = 0, although some deviation from zero will be observed [Panel (b) in Fig. 5]. Third, when the two methods are somewhat dissimilar, the overlap between the two neighbor lists will be small, forming a broader NPI distribution [Panel (c) in Fig. 5].

**Fig. 6:**
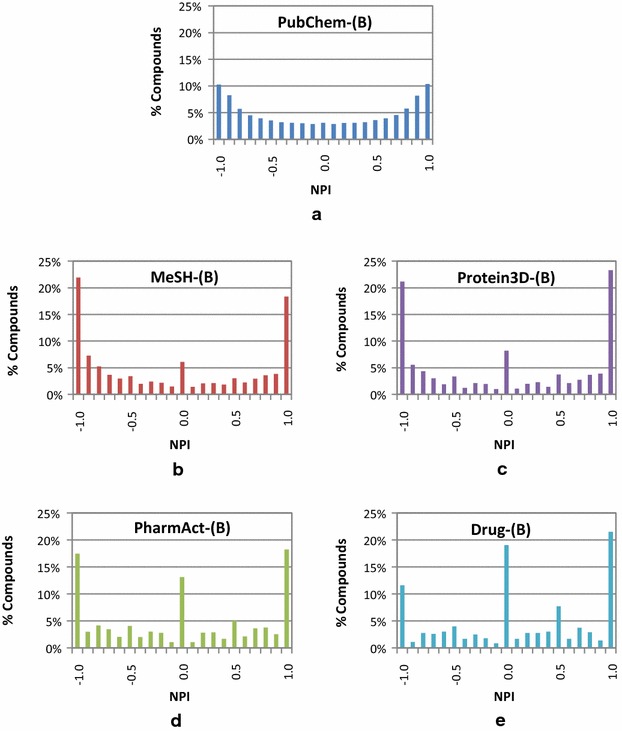
Binned distribution of the neighbor preference indices (NPIs) for compounds in the five data tests in Series B. The binned distribution (in 0.1 increments) of the NPI values for the PubChem-(B) set has a bimodal shape with two maxima at NPI = ±1 and a minimum at NPI = 0. For the other four sets, the maxima at NPI = ± 1 became more prominent and an additional maximum appeared at NPI = 0, progressing from **a** to **e**

Figure 6 shows the distribution of the NPIs for compounds in the five Series B data sets. The shape of the NPI distribution for the PubChem-(B) set does not look like any of the scenarios hypothesized in Fig. 5. This discrepancy arises from an invalid assumption underlying all three hypotheses that structural similarity among the majority of the compounds in the data set can readily be recognized by both neighboring schemes. In reality, as shown in Panel (a) of Fig. 6, the structural similarities that PubChem 2-D and 3-D neighborings recognize are very different from each other. For example, 2-D neighboring does not recognize the similarity of 10% of the PubChem-(B) compounds with other compounds that 3-D neighboring recognizes [i.e., NPI ≅ −1, corresponding to the left-most column in Fig. 6a]. On the contrary, 3-D neighboring does not recognize the similarity of another 10% of PubChem-(B) compounds with their 2-D neighbors [i.e., NPI ≅ 1, corresponding to the right-most column in Fig. 6a]. Compounds whose similarity can be recognized equally well by both neighborings correspond to zero on the NPI distribution curve of the PubChem-(B) set. These observations further indicate a substantial degree of the complementariness between the two PubChem neighboring methods.

Interestingly, the shape of the NPI histograms for the other data sets in Series B [i.e., Mesh-(B), Protein3D-(B), PharmAct-(B), and Drug-(B)] are different from that of the PubChem-(B) set. The outermost columns at each end of the histograms become more prominent, indicating that the fraction of compounds with extreme NPIs (i.e., those with NPI values of −0.95 to −1.00 and those with +0.95 to +1.00) increased in these subsets. In addition, the fraction of compounds with an NPI between −0.05 and 0.05 increases as the size of the data set decreases. This indicates that these subsets of the PubChem-(B) set do not well represent the chemical space covered by the PubChem-(B) set. Note that these four subsets were generated by checking whether compounds have a particular type of annotation (for example, whether a compound has been prominently mentioned in a biomedical journal article, whether it has been co-crystalized with a protein target, whether it has a known pharmacological action, or whether it is a known active drug ingredient). In other words, the four subsets correspond to four narrowly focused subspaces of the PubChem-(B) data set and, unlike the overall chemical set, may be dominated by closely related analogues and structurally similar scaffolds in an attempt to identify similar bioactivity.

### Data set dependency of neighbor preference indices

**Fig. 7:**
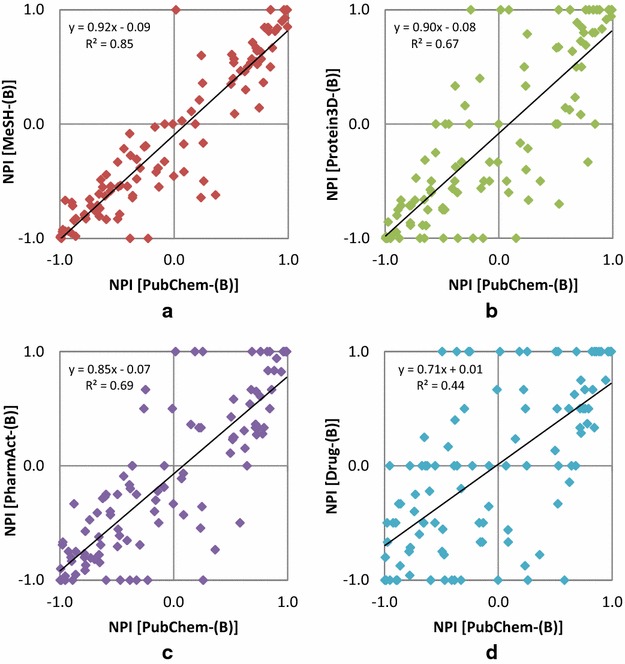
Comparison of the NPI values of 108 compounds for the MeSH-(B) (**a**), Protein3D-(B) (**b**), PharmAct-(B) (**c**), and Drug-(B) (**d**) sets with those for the PubChem-(B) set. The correlation of the NPI values with the PubChem-(B) set data is largest for the MeSH-(B) set and smallest for the Drug-(B) set

 Investigating the data set dependency of NPI values for a given compound requires a set of compounds that are contained in all five data sets, but only 108 compounds occurred in all the five data sets. This is primarily because many drug molecules in the Drug-(B) set were not contained in the Protein-(B) set due to the lack of their experimentally determined protein-bound structures. While this set is not large enough to represent all chemical space, NPI values of these compounds still provides some insights on the data set dependency of NPI values. Figure 7 compares the NPI values of these 108 compounds for the PubChem-(B) set with those for the other data sets in Series B. The NPI values for the MeSH-(B) set show a linear correlation with those for the PubChem-(B) set, with an R^2^ value of 0.85. However, the NPI value correlation with the PubChem-(B) set was weaker for the other three subsets. Especially, the R^2^ value for the Drug-(B) set was as small as 0.44.

**Fig. 8:**
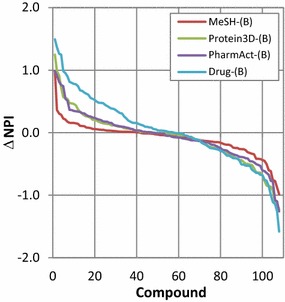
Comparison of the NPI values of 108 compounds for the MeSH-(B), Protein3D-(B), PharmAct-(B), and Drug-(B) sets with those for the PubChem-(B) set. A positive ΔNPI value for a given data set indicates that a compound has a stronger 2-D neighbor preference in that data set than in the PubChem-(B) set. On the contrary, a negative ΔNPI value indicates a stronger 3-D neighbor preference

**Fig. 9:**
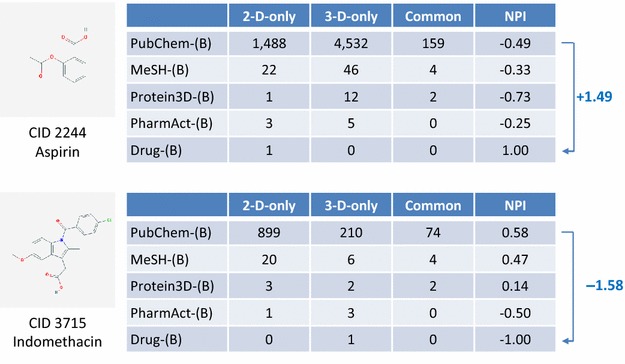
The numbers of neighbors and Neighbor Preference Indices (NPIs) of CID 2244 (aspirin) and CID 3715 (indomethacin). Among the 108 compounds that are common to the five Series B data sets, CID 2244 and CID 3715 show the largest difference in the Neighbor Preference Index (NPI) between the PubChem-(B) and Drug-(B) sets. Note that the NPI values of the compounds for the PubChem-(B) set have opposite signs to those for the Drug-(B) sets

The differences in the NPI values of the 108 compounds between the PubChem-(B) set and the four annotated sets are plotted in Fig. 8. In general, the magnitude of the NPI value difference (ΔNPI) from the PubChem-(B) set increases as the data set becomes smaller. The increase in 2-D similarity preference (with positive ΔNPI values) is more prominent than the increase in 3-D similarity preference (with negative ΔNPI values). Figure 9 shows the numbers of neighbors of aspirin (CID 2244) and indomethacin (CID 3715), respectively, which shows the largest NPI changes for the Drug-(B) set in each direction (corresponding to each end of the Drug-(B) curve in Fig. 8).

Aspirin has 1488 2-D-only neighbors and 4532 3-D-only neighbors in the PubChem-(B) set, resulting in an NPI value of −0.49. However, it has an NPI value of 1.00 in the Drug-(B) set, with one 2-D-only neighbor (CID 5161; salicylsalicylic acid) and no 3-D-only neighbors. On the other hand, indomethacin has an NPI value of +0.58 for the PubChem-(B) set, with 899 2-D-only neighbors and 210 3-D-only neighbors, but it has only one 3-D neighbor in the Drug-(B) set, resulting in an NPI value of −1.00. Note that the signs of the NPI values of the two compounds for the PubChem-(B) set are opposite to those for the Drug-(B) set. This indicates that the nature of the chemical spaces spanned by the two sets is very different in terms of which neighboring scheme is better in recognizing structural similarity to aspirin and indomethacin.

**Fig. 10:**
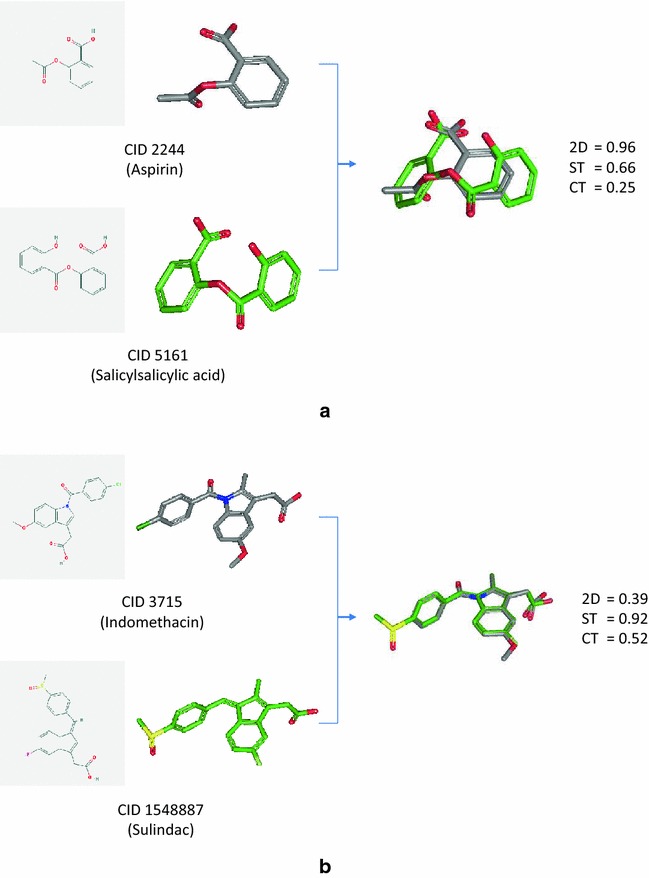
Complementarity between 2-D and 3-D neighborings. 2-D and 3-D similarity scores **a** between CID 2244 (aspirin) and CID 5161 (salicylsalicylic acid) and **b** between CID 3715 (indomethacin) and CID 1548887 (sulindac). For both cases, one neighboring method can recognize structural similarity that the other method cannot

As shown in Fig. 10, salicylsalicylic acid corresponds to substitution of a phenyl (C_6_H_5_ group) group for the methyl group in aspirin, and the 2-D Tanimoto between the two compounds was 0.96, which is above the PubChem 2-D neighboring threshold of 0.9. However, their shape-Tanimoto (ST) and color-Tanimoto (CT) scores were 0.66 and 0.25, respectively, less than the 3-D neighboring criteria. On the contrary, the 2-D Tanimoto between indomethacin and sulindac was as small as 0.39, but their ST and CT scores were 0.92 and 0.52, respectively, which are greater than 3-D neighboring thresholds. The examples in Fig. 10 illustrate how the two neighboring schemes can complement each other, in that one neighboring method recognizes the structural similarity that the other method cannot recognize.

### Effects of stereochemistry upon 2-D and 3-D neighborings

The PubChem fingerprints used for 2-D similarity evaluation in PubChem do not take into account stereochemistry of molecules (such as cis–trans isomerism and chirality). Therefore, different stereo isomers that have the same molecular formula and atom connectivity are represented with the same fingerprint, regardless of whether the configuration stereo centers are explicitly defined or not. For example, both (E) and (Z) forms of 1,2-dichloroethene (CID 638186 and CID 643833) have the same fingerprint as 1,2-dichloroethene (CID 10900). As a results, 2-D similarity evaluation between these stereo isomers always yields a Tanimoto score of “1.0”, classifying them as neighbors of each other. In addition, 2-D neighboring of these stereo isomers results in the same set of 2-D neighbors.

On the contrary to the 2-D neighboring, PubChem 3-D neighboring is not blind to stereochemistry because it uses 3-D conformer models that take stereochemistry into account. PubChem generates a different 3-D conformer model for a given stereo isomer. The conformer model for a compound with unspecified stereo centers is constructed by generating conformers for each stereo isomer arising from enumeration of the undefined stereo centers, and then combining them together [22]. As a result, the use of different conformers for 3-D neighboring of stereo isomers may yield different sets of 3-D neighbors, as discussed in our previous paper [7].

## Conclusions

The overlap between PubChem 2-D and 3-D similarity neighboring approaches were analyzed as a function of annotation type, using ten data sets: five data sets in Series A [i.e., PubChem-(A), MeSH-(A), Protein3D-(A), PharmAct-(A), and Drug-(A)] and five data sets in Series B [i.e., PubChem-(B), MeSH-(B), Protein3D-(B), PharmAct-(B), and Drug-(B)]. The five data sets in Series A considered all compounds [PubChem-(A)], those prominently mentioned in a biomedical journal article [MeSH-(A)], those found in a protein–ligand complex crystal structure [Protein3D-(A)], those with a known pharmacological action [PharmAct-(A)], and those which are approved drugs [Drug-(A)]. A direct comparison between PubChem 2-D and 3-D neighbors using the Series A data sets revealed a bias towards 2-D neighbors as 3-D neighboring does not consider salts and mixtures. To remove this bias, the Series B data sets were generated by considering only parent compounds (in effect discarding salts and mixtures) with a computed 3-D description in PubChem. For both PubChem-(A) and PubChem-(B) sets, the overlap between 2-D and 3-D neighbors were only about 2% of the total neighbors. In other words, the PubChem 2-D and 3-D similarity approaches are nearly orthogonal. Considering the debate over 2-D and 3-D similarity methods [29–34], this is a surprising finding. For the subsets containing compounds with specific types of annotation, the overlap increased substantially as the data sets became smaller. However, it did not exceed 31% [for the Drug-(B) set] and substantial fractions of neighbors were still either 2-D-only or 3-D-only.

To further investigate complementarity between 2-D and 3-D neighborings, the NPI of a molecule for a given data set was introduced that quantifies whether a molecule has more 2-D or 3-D neighbors. The NPI histograms for the PubChem-(B) set shows a bimodal shape with two maxima at NPI = ±1 and a minimum at NPI = 0. It indicates that, for the majority of the compounds in PubChem, their structural similarity to other compounds can be recognized only by either of the 2-D or 3-D neighborings, but not by both. Therefore, considering both 2-D and 3-D approaches in PubChem appears to be beneficial.

Interestingly, the shape of the NPI value histogram for the PubChem-(B) set is not similar to those for its four subsets [i.e., MeSH-(B), Protein3D-(B), PharmAct-(B), and Drug-(B)]. The NPI value histograms show a more polarized trimodal profile with a greater fraction of compounds with a strong preference for one neighboring method over the other (at NPI = ±1) as well as compounds with a neutral preference (at NPI = 0) but less so in between the extremes. As such, one would be well advised to use both 2-D and 3-D similarity when searching for chemicals that are well studied.

The results of our study show the complementarity between the 2-D and 3-D neighbors in PubChem. Each neighboring approach can identify structural similarity that the other neighboring approach cannot detect. Put in other words, they appear to have equal value to interrelate chemical structures with similar counts of neighbor pairs by each. Depending on use case (such as looking for analogues of chemicals in a series or interrelating chemical series), scientists may prefer to use one approach over the other or both to retrieve information on chemicals similar to a compound of interest.

## Methods

### Data sets

#### Series A data sets

The present study employed ten different data sets (five data sets each for two series: A and B). The number of compounds per data set is listed in Table 1. The PubChem-(A) set represents the entire chemical space spanned by compounds stored in PubChem [1] whose CID is less than or equal to 60,182,254, reflecting those CIDs with both 2-D and 3-D neighboring data available at the time of analysis. Note that, while 2-D neighbors are updated on a daily basis, 3-D neighbors are updated less frequently because 3-D neighboring is more CPU-intensive. As a result, newly added compounds in PubChem may be considered in 2-D neighboring sooner than 3-D neighboring. Therefore, the use of a CID cut-off allows for a more direct comparison of 2-D and 3-D neighbor counts for the purpose of this study.

PubChem is integrated with the Entrez system [5], the primary search engine of NCBI. The Entrez interface provides filters that can restrict a search to a particular compound subset in PubChem. The Entrez filters below were used to generate the four different subsets of PubChem-(A):

1. pccompound_mesh for MeSH-(A): this filter includes compounds that have a link to the MeSH database [25]. MeSH (Medical Subject Headings) is the NLM controlled vocabulary thesaurus, and is used to index PubMed citations. The chemicals with a MeSH link have been mentioned in the biomedical literature on several occasions and are deemed (by human curators) to be of sufficient importance to be added to MeSH. The MeSH links are generated using PubChem chemical name matching approaches.
2. pccompound_structure for Protein3D-(A): this filter includes compounds found in the Molecular Modeling Database (MMDB) [27]. The MMDB contains experimentally resolved structures of proteins, RNA and DNA, derived from the Protein Data Bank (PDB) [26], including information about small molecule ligands bound to macromolecule structures. Therefore, the Protein3D-(A) set contains the compounds whose macromolecule-bound 3-D experimental structure is available.
3. pccompound_mesh_pharm for PharmAct-(A): this filter includes compounds that have a pharmacological action link in the MeSH database, indicating that the biological role of the chemical is known. Note that PharmAct-(A) is a subset of MeSH-(A).
4. pccompound_drugs for Drug-(A): this filter limits compounds to those that are known drugs as defined by the PubChem integration of the NLM DailyMed [28] resource. The information content of DailyMed is provided by the U.S. Food and Drug Administration (FDA) and includes structured product labelling (SPL) drug information submitted by drug companies who manufacture and sell them.

These filters allow users to obtain CIDs that have particular annotation types. For example, CIDs with the “drug” annotation can be retrieved via the URL: https://www.ncbi.nlm.nih.gov/pccompound/?term=pccompound_drugs[filter].

#### Series B data sets

The present study attempts to compare the ability of PubChem 2-D and 3-D neighborings to interrelate chemicals that have a particular annotation type in common. Two primary issues are apparent in the analysis of the Series A data sets. First, not all compounds in the Series A data sets have the necessary computed 3-D conformer models required for 3-D neighboring, as the PubChem3D project covers only about 90% of the compound records in PubChem [6, 18, 23], excluding by design multi-component structures like salts. Second, it is not uncommon for annotation to be attributed to a salt form as opposed to the primary active component (see Fig. 1 for an example).

To address these two issues, Series B data sets were generated by including the “parents” of all the compounds in the Series A data sets and then by selecting only those resulting structures with an available computed 3-D conformer description. To achieve this, NCBI’s FLink [35] was used with the PubChem Compound Entrez filters “pccompound_pccompound_parent” and “has_3d_conformer” to retrieve the parent compounds and the structures with a computed 3-D description, respectively. PubChem defines the “parent” of a mixture as the carbon-containing component whose heavy atom count is ≥70% of the sum of the heavy atom counts of all unique covalent units [1]. The parent compound is neutralized through modification of its protonation state during the PubChem standardization process [1]. Because PubChem3D does not compute conformer models for compound records with multiple covalent units [6, 18, 23], all of the resulting compounds in the Series B data sets are single-component compounds with computed 3-D conformer descriptions. The sizes of the data sets in both Series A and B are compared in Table 1.

### PubChem 2-D and 3-D neighboring relationships

The PubChem 2-D and 3-D neighboring processes are briefly described below. More detailed description is given elsewhere [6, 7].

#### PubChem “Similar Compounds” 2-D neighboring

The PubChem substructure fingerprints [8] are 881-bit-long binary (0/1) vectors, each bit of which represents the absence (0) or presence (1) of a particular structural characteristic found in a chemical structure, such as an element count, a type of ring system, atom pairing, atom environment (nearest neighbors), and so on. A more detailed description of this fingerprint system is available in Ref. [8]. The PubChem fingerprints are used to quantify 2-D similarity between two chemical structures in PubChem, in conjunction with the Tanimoto coefficient [9–11], defined as the following equation:

1 $$Tanimoto = \frac{{N_{AB} }}{{N_{A} + N_{B} - N_{AB} }}$$

where *N*_*A*_ and *N*_*B*_ are the counts of bits set in the fingerprints representing molecules A and B, respectively, and *N*_*AB*_ is the count of common bits set in both fingerprints. A Tanimoto coefficient ranges from 0 (for no similarity between molecules) to 1 (for identical molecules, relative to the resolution of the substructure fingerprint).

PubChem 2-D neighboring quantifies molecular similarity using the PubChem substructure fingerprints and Tanimoto coefficient as described above. If two chemical structures in PubChem have a Tanimoto score of 0.9 or greater, they are considered as “Similar Compound” 2-D neighbors of each other.

#### PubChem “Similar Conformers” 3-D neighboring

PubChem 3-D neighboring is described in detail elsewhere [6, 7]. It quantifies molecular similarity using the Gaussian-shape overlay method by Grant and Pickup [12–15], implemented in ROCS [16, 17]. In this approach, molecular shape is described with an atom-centered Gaussian function, which allows for a rapid shape superposition, compared to hard sphere volume approaches. Recent studies [36–38] show that this method can be comparable with, and often better than, structure-based approaches in virtual screening, both in terms of overall performance and consistency.

This 3-D similarity method [12–14, 16, 17] considers two aspects of molecular similarity: shape similarity and feature similarity. The shape similarity [12, 14, 16, 17, 39] between molecules is quantified with the shape-Tanimoto (ST), which is defined as the following:

2 $$ST = \frac{{V_{AB} }}{{V_{AA} + V_{BB} - V_{AB} }}$$

where *V*_*AA*_ and *V*_*BB*_ are the self-overlap volumes of molecules A and B, respectively, and *V*_*AB*_ is the overlap volume between molecules A and B. The feature similarity [17, 39], which is the similarity of 3-D orientation of protein-binding “features” between conformers, is evaluated by checking the overlap of “fictitious” feature atoms (also called “color” atoms) that represent six types of functional groups including hydrogen bond donors and acceptors, cations, anions, hydrophobes, and rings. Its quantification uses the color-Tanimoto (CT) [17, 39], given by the following equation:

3 $$CT = \frac{{\sum\nolimits_{f} {V_{AB}^{f} } }}{{\sum\nolimits_{f} {V_{AA}^{f} } + \sum\nolimits_{f} {V_{BB}^{f} } - \sum\nolimits_{f} {V_{AB}^{f} } }}$$

where the index “*f*” indicates any of the six feature atom types, $V_{AA}^{f}$ and $V_{BB}^{f}$ are the self-overlap volumes of conformers A and B for feature atom type *f*, respectively, and $V_{AB}^{f}$ is the overlap volume between conformers A and B for feature atom type *f*. To consider the steric shape similarity and chemical feature similarity simultaneously, the combo-Tanimoto (ComboT) is introduced, as specified by the following equation:

4 $$ComboT = ST + CT$$

Because both ST and CT scores range from 0 (for no similarity) to 1 (for identical molecules), by definition, the ComboT score can have a value from 0 to 2 (without normalization).

These three metrics can be computed at two different conformer superpositions: (1) the shape-optimized (or ST-optimized) superposition, where the shape overlap between the two conformers is maximized, and (2) the feature-optimized (or CT-optimized) superposition, where both the shape and feature are considered simultaneously to find the best superposition between the conformers. As a result, PubChem3D quantifies 3-D molecular similarity using six different scores: ST, CT, and ComboT scores for each of the superposition methods. However, because PubChem 3-D neighboring uses the ST-optimized scores only, all the ST, CT, and ComboT scores mentioned in this paper refer to the ST-optimized scores, unless otherwise indicated.

If any of the conformer pairs arising from a pair of two compounds has a ST score of ≥0.8 and a CT score of ≥0.5, those compounds are considered to be neighbors of each other. This guarantees that there is at least one conformer pair with a ComboT score ≥1.3 for each pair of compounds that are neighbors.

### Conformer models for 3-D neighboring

PubChem 3-D neighboring requires a computed 3-D conformer model for each compound considered. These conformer models were generated using the OMEGA software from OpenEye Scientific Software, Inc., as described in more detail elsewhere [6, 18, 23]. While these conformer models contain up to 500 sampled conformers for each compound, many of the PubChem3D services support only up to ten conformers per compound. To ensure that the conformers employed represent the overall diversity of shape and feature of a given molecule, PubChem3D computes a diverse conformer ordering. This conformer ordering provides guidance on what conformers to choose when only a subset of the conformers available in a conformer model are used for 3-D similarity comparison.

Despite the use of various filtering schemes to improve its speed [7, 20], 3-D neighboring is not fast enough to consider all possible conformers for each compound. The initial PubChem3D neighboring started a few years ago using a single conformer per compound and it has been gradually extended to more diverse conformers per compound. Up to ten conformers per compound will be considered in the future. The neighboring results used in the present study were from five diverse conformers per compound, as available in PubChem as of January 2013.

### Definition of Neighbor Preference Index

The Neighbor Preference Index (NPI) of a compound quantifies which of the two neighboring methods can identify more chemical structures similar to that compound in a given data set. It is defined as the following equation:

5 $$NPI = \frac{{N_{2 - D - only} - N_{3 - D - only} }}{{N_{total} }}$$

where *N*_*2*-*D*-*only*_ and *N*_*3*-*D*-*only*_ are the numbers of 2-D-only and 3-D-only neighbors of the compound, and *N*_*total*_ is the total number of the neighbors of the compound. An NPI value can range from −1 (when all neighbors are 3-D-only neighbors) to +1 (when all neighbors are 2-D-only neighbors). An NPI value of zero indicates that there is no preference for one method over the other (i.e., the number of 2-D neighbors is equal to the number of 3-D neighbors). Although the number of common neighbors (which are 2-D and 3-D neighbors simultaneously) does not explicitly appear in the equation above, it implicitly contributes to the NPI through *N*_*total*_. That is, the NPI value approaches zero as the number of common neighbors increases. Note that neighboring of different data sets results in different sets of neighbors for a given compound. Therefore, the NPI value of a compound also depends on the nature of the data set to which it belongs (i.e., what compounds the data set has).

Note that, because the choice of neighboring thresholds affects the neighbor counts of a given compound and hence its NPI value, the use of NPI values for comparing the two neighboring methods requires that the neighboring thresholds employed be comparable. Therefore, given that the two PubChem neighboring approaches are established with thresholds that are unlikely to change, it is worthwhile to consider the statistical basis of the two PubChem similarity methods.

A recent study [22] shows that the average and standard deviation of the 2-D and 3-D similarity scores are 0.42 ± 0.13 and 0.77 ± 0.13, respectively, for randomly selected biologically tested compounds in PubChem. The 3-D similarity statistics are for the ST-optimized ComboT score [Eq. (4)] of a compound–compound pair, which is the highest ComboT score among those of all conformer pairs arising from the compound pair (computed using ten diverse conformers per compound). While not exactly the same as PubChem 3-D neighboring, these 3-D statistics should be considered as a lower-bound, with PubChem 3-D neighboring further restricted (and being more exclusive) by statistically more-significant thresholds of ST ≥0.8 *and* CT ≥0.5 (and a minimum ComboT ≥1.3). These statistics suggest that the 2-D and 3-D neighboring thresholds are 3.7 and 4.1 standard deviations away from the random average values, respectively [i.e., 3.7 = (0.90 − 0.42)/0.13 for 2-D similarity and 4.1 = (1.3 − 0.77)/0.13 for 3-D similarity]. This translates into a probability of two random structures in PubChem being 2-D neighbors and 3-D neighbors as 0.0111% (1 in 9000) and 0.00228% (1 in 43,825), respectively. In addition, it suggests that the two neighboring thresholds are comparable (within a factor of five of each other, i.e., 4.86 = 0.0111%/0.00228%), with a small bias towards 2-D neighbors. Lastly, it also suggests that the thresholds are suitably high to limit chance correlations of neighbors.
